# CD8^+^CD122^+^ T-Cells: A Newly Emerging Regulator with Central Memory Cell Phenotypes

**DOI:** 10.3389/fimmu.2015.00494

**Published:** 2015-10-19

**Authors:** Junfeng Liu, Dacan Chen, Golay D. Nie, Zhenhua Dai

**Affiliations:** ^1^Section of Immunology, Division of Dermatology, Second Affiliated Hospital, Guangdong Provincial Academy of Chinese Medical Sciences, Guangzhou University of Chinese Medicine, Guangzhou, China; ^2^School of Medicine, University of Texas Medical Branch (UTMB), Galveston, TX, USA

**Keywords:** regulatory T-cells, CD8^+^CD122^+^ T-cells, immunoregulation, allograft survival, transplant tolerance, memory T-cells

## Abstract

CD8^+^CD122^+^ T-cells have been traditionally described as antigen-specific memory T-cells that respond to previously encountered antigens more quickly and vigorously than their naïve counterparts. However, mounting evidence has demonstrated that murine CD8^+^CD122^+^ T-cells exhibit a central memory phenotype (CD44^high^CD62L^high^), regulate T cell homeostasis, and act as regulatory T-cells (Treg) by suppressing both autoimmune and alloimmune responses. Importantly, naturally occurring murine CD8^+^CD122^+^ Tregs are more potent in immunosuppression than their CD4^+^CD25^+^ counterparts. They appear to be acting in an antigen-non-specific manner. Human CD8^+^CXCR3^+^ T-cells are the equivalent of murine CD8^+^CD122^+^ Tregs and also exhibit central memory phenotypes. In this mini-review article, we will summarize recent progresses in their phenotypes, homeostatic expansion, antigen-specificity, roles in the suppression of alloimmune and autoimmune responses, and the mechanisms underlying their inhibitory function.

## Introduction

Upon reencounter with previously recognized antigens, memory T-cells respond more rapidly and vigorously than their naïve counterparts. They are somewhat resistant to immunosuppression, such as costimulatory blockade (1, 2). Memory T-cells can rapidly trigger alloimmune responses through the production of various inflammatory cytokines (3). Previous studies have also shown that early infiltration of CD8^+^ memory T-cells into allografts, including hearts, kidneys, and livers, facilitates allograft rejection and presents a hurdle to achieving long-term allograft survival (4–6). Therefore, they are generally considered as a major barrier to long-term allograft survival or tolerance (7). In particular, CD8^+^CD122^+^ T-cells have previously been described as antigen-specific memory T-cells (8–10). However, emerging evidence has shown that central memory CD8^+^CD122^+^ T-cells (CD44^high^CD62L^high^) also play a role in regulating T cell homeostasis and serve as regulatory T-cells (Tregs). Since original studies revealed that CD8^+^ T-cells exhibit Treg properties (11–13), more and more studies have confirmed their immunosuppressive activities. Suzuki’s research group has provided the early evidence that CD8^+^CD122^+^ T-cells maintain T cell homeostasis (14), whereas more recent studies have suggested that CD8^+^CD122^+^ T-cells are Tregs that suppress conventional T cell responses (14–20) and control autoimmune diseases (21, 22). We have found that memory CD8^+^CD122^+^ T-cells as well as bystander central memory CD8^+^ T-cells are also Tregs that inhibit murine allograft rejection (23, 24). Moreover, others have shown that central memory CD8^+^ T-cells mediate lung allograft acceptance (25). Therefore, CD8^+^CD122^+^ Tregs may correspond to their CD4^+^CD25^+^FoxP3^+^ counterparts since CD122 is β subunit of IL-2 receptor, whereas CD25 is an α subunit of the same receptor on T cells (26). More importantly, we have recently demonstrated that CD8^+^CD122^+^ Tregs are more potent in their suppression of allograft rejection than their CD4^+^CD25^+^ counterparts (27). Hence, not only are memory CD8^+^CD122^+^ T-cells Tregs but can also boost the strength of Treg-mediated regulation.

Although CD8^+^CD122^+^ Tregs may regulate immune responsiveness by production of IL-10, TGFβ1, and IFNγ, the exact mechanisms underlying their suppression are still largely unknown. More studies are required to fully understand their mechanisms and clearly distinguish between effectors and regulators that originate from memory CD8^+^CD122^+^ T-cells, although PD-1 expression recently has been shown to define CD8^+^CD122^+^ T-cells as the memory versus Tregs (24). In this mini-review article, we will briefly summarize new progresses in their phenotypes, antigen specificity, roles in the suppression of alloimmune and autoimmune responses, and immune mechanisms underlying their inhibitory function.

## CD8^+^CD122^+^ Treg Phenotypes

Mounting evidence has suggested that CD8^+^CD122^+^ T-cells are Tregs, which can suppress both autoimmunity and alloimmunity. They exhibit memory-like T cell phenotypes with regulatory functions. Murine CD8^+^CD122^+^ Tregs express CD122 (IL-2Rβ) and CXCR3, but not CD25, whereas their CD4^+^CD25^+^ counterparts do not express CD122. CD8^+^CD122^+^ Tregs are also CD44^high^, CD62L^high^ CCR7^+^, and largely CD127^−^ (24, 28). However, CD8^+^CD122^+^ Tregs are FoxP3-negative (24), indicating that they are a different subset from inducible CD8^+^FoxP3^+^ Tregs (29). Memory-like CD8^+^CD122^+^ T-cells, which also express CD38, suppress CD4^+^ T-cell activation (30), whereas another subset of CD8^+^ Tregs carry both CD122 surface marker and the class-I MHC receptor Ly49 (21). We have previously shown that PD-1 expression defines CD8^+^CD122^+^ T-cells as Tregs versus memory T-cells (24).

Human equivalent of murine CD8^+^CD122^+^ Tregs has also recently been characterized. They exhibit an *in vitro* suppressive property with phenotypes of CD8^+^CXCR3^+^CD45RA^−^ and largely CD62L^+^ or CCR7^+^, but do not express CD122 (31). However, it remains to be defined if CXCR3 expression alone can distinguish memory from regulatory human CD8^+^ T-cells since effector CD8^+^ T-cells may also express CXCR3. Taken together, both murine CD8^+^CD122^+^ and human CD8^+^CXCR3^+^ Tregs exhibit the phenotypes of central memory T-cells.

## Age-Associated Distribution and Naturally Arising Features of Murine CD8^+^CD122^+^ Tregs

The percentages of CD122^+^ T-cells within CD8^+^ population fluctuate between 10 and 50%, depending on mouse age. Overall, the percentages of CD122^+^ T-cells in CD8^+^ population are very high in young mice, reduced to the lowest level of ~10% at the age of 8–10 weeks, and then increased with aging, exhibiting a pattern of two age-related phases (14). A high rate of CD8^+^CD122^+^ T-cells in young mice may represent a regulatory cell component since previous studies and ours have proved that these cells derived from young mice are indeed Tregs, whereas the increased number of CD8^+^CD122^+^ T-cells at old age could be due to the increase in memory CD8^+^CD122^+^ T-cells. It remains to be determined if the latter are regulatory or memory T-cells and if they contain both components. On the other hand, CD8^+^CD122^+^ T-cells that are proved to be Tregs have been isolated, to our knowledge, from naïve mice, suggesting that they are indeed naturally arising cells. However, it is unknown whether CD8^+^CD122^+^ Tregs can be induced *in vitro* and *in vivo*. It is also unclear if they exhibit the same phenotypes upon expansion *in vitro* or *in vivo*. These questions warrant further investigations given that CD8^+^CD122^+^ Tregs are more efficient but yet less studied than conventional CD4^+^CD25^+^ Tregs.

## Homeostasis and Expansion of Memory-Like CD8^+^CD122^+^ Tregs

IL-15 is essential for the generation and survival of memory CD8^+^ T-cells (32, 33) while administration of recombinant IL-15 significantly augments their numbers (8, 34). We have demonstrated that administration of IL-15 expands adoptively transferred CD8^+^CD122^+^ Tregs and enhances their suppression of allograft rejection (27). We have also found that IL-15 promotes their expansion *in vitro* as well, suggesting that IL-15 provides a critical signal that drives homeostatic expansion of memory-like murine CD8^+^CD122^+^ Tregs. However, it remains to be defined if IL-15 also drives homeostatic expansion of human CD8^+^CXCR3^+^ Tregs. IL-15 may not do so since they do not express CD122. In order to enhance Treg suppression in a clinic setting, further studies are warranted to identify signals that are required for homeostatic expansion of human CD8^+^CXCR3^+^ Tregs.

Since IL-15 can expand both memory CD8^+^ T-cells and CD8^+^CD122^+^ Tregs, it may act as a double-edged sword in the face of an allograft. It would be difficult to use IL-15 to promote CD8^+^CD122^+^ Treg-mediated graft acceptance without expanding memory CD8^+^ T-cells. Recent studies have shown that administration of IL-15 enhances anti-tumor immunity when blocking CD8^+^CD122^+^ Treg function (35). In those studies, IL-15 enhanced memory CD8^+^ T-cell function, but not CD8^+^CD122^+^ Treg suppression, given that Tregs were inhibited by PD-1 signaling blockade. In a transplant setting, however, we need to boost the Treg suppression. We have previously succeeded in utilizing low doses of IL-15 to promote adoptively transferred CD8^+^CD122^+^ Treg suppression of allograft rejection (27), indicating that when CD8^+^CD122^+^ Tregs are transferred in large numbers, IL-15 likely promotes the expansion of more CD8^+^CD122^+^ Tregs than endogenous memory CD8^+^ T-cells.

## Is CD8^+^CD122^+^ Treg-Mediated Suppression Antigen-Specific?

It remains to be defined if the immunosuppression mediated by murine CD8^+^CD122^+^ Tregs is antigen specific. Recent studies have shown that CD8^+^CD122^+^ Tregs exhibit CDR3 sequences of T-cell receptor β chain (36), and that the distribution of the CDR3 length is following a Gaussian-like one, except for Vb13. Based on this original study, it is possible that murine CD122^+^CD8^+^ T-cells are not selected in the thymus. Moreover, we have demonstrated that bystander memory CD8^+^ T-cells with central memory phenotypes can inhibit allograft rejection in an antigen-non-specific manner (23). We have also shown that CD8^+^CD122^+^ Treg-mediated suppression *in vitro* is donor-non-specific (24), supporting a finding by others that memory-like CD8^+^CD38^+^ T-cells suppress CD4^+^ T cell activation in an antigen-independent fashion (30). Therefore, it is possible that their suppression of immune responses is not antigen specific.

## CD8^+^CD122^+^ Tregs Inhibit Immune Responses in Animal Models of Diseases

Early evidence has shown that CD8^+^CD122^+^ Tregs regulate T cell homeostasis (14), whereas recent studies have suggested that they play an important role in the suppression of autoimmune responses. Transfer of CD8^+^CD122^+^ Tregs significantly improved clinical symptoms of experimental autoimmune encephalomyelitis (EAE) (37), whereas IL-15-dependent CD8^+^CD122^+^ Tregs also ameliorated EAE by suppressing IL-17 production (38). Dendritic cells (DCs) expressing B7-H1 molecule recruited CD8^+^CD122^+^ Tregs, which in turn suppressed the onset of EAE (39). CD8^+^CD38^+^CD122^+^ Tregs inhibited effector CD4^+^ T-cell activation and mitigated murine EAE by delaying the disease occurrence (30). Studies using HLA-DR3 transgenic mice also demonstrated that CD8^+^CD122^+^ T-cells could regulate EAE (22). Depleting CD8^+^CD122^+^ T-cells augmented the incidence of autoimmune Graves’ hyperthyroidism (40). Interestingly, CD8^+^CD122^+^ and CD4^+^CD25^+^ Tregs cooperatively suppressed colitis mediated by CD4^+^ T-cells (19), whereas the systemic lupus erythematosus-like disease in B6-Yaa mutant mice was associated with a defect in CD8^+^CD122^+^ T-cells (21). Therefore, it has been clear that CD8^+^CD122^+^ Tregs play an essential role in the suppression of various experimental autoimmune diseases.

Emerging evidence has shown that memory-like CD8^+^CD122^+^ Tregs can also suppress alloimmune responses and allograft rejection. We have unveiled the first evidence that CD8^+^CD122^+^ Tregs suppress allograft rejection, whereas their PD-1^+^ component is more efficient than unfractionated CD8^+^CD122^+^ Treg population (24). Therefore, both CD4^+^CD25^+^ and CD8^+^CD122^+^ T-cells are Tregs that can inhibit allograft rejection. In order to seek more efficient Treg suppression for potential clinical applications, we determined the efficacy of CD8^+^CD122^+^ versus CD4^+^CD25^+^ Tregs in their suppression of allograft rejection. We found that CD8^+^CD122^+^ Tregs were actually more potent in suppression of allograft rejection and underwent faster homeostatic proliferation *in vivo* than their CD4^+^CD25^+^ counterparts (27). Moreover, they also were more effective in the suppression of *in vitro* T cell proliferation than their CD4^+^CD25^+^ counterparts. Importantly, the adoptive transfer of CD8^+^CD122^+^, but not CD4^+^CD25^+^, Tregs plus treatments with recombinant murine IL-15 significantly extended allograft survival even in immune competent wild-type mice (27). By contrast, the adoptive transfer of CD4^+^CD25^+^ Tregs, together with administration of recombinant IL-15, did not significantly suppress allograft rejection. We postulated that administering IL-15 promoted the expansion of transferred CD8^+^CD122^+^ Tregs, which in turn extended allograft survival. Krupnick et al. found that costimulatory blockade-mediated long-term allograft acceptance was dependent on the rapid infiltration of the lung graft by central memory CD8^+^ T-cells and on their production of IFNγ, which in turn induced nitric oxide (NO) and inhibited alloimmune responses (25). Taken together, CD8^+^CD122^+^ Tregs with central memory phenotypes can regulate both autoimmunity and alloimmunity, suggesting an important role for CD8^+^CD122^+^ Tregs in the modulation of pathogenic immune responses.

## Mechanisms Responsible for Immunosuppression Mediated by CD8^+^CD122^+^ Tregs

**Figure 1:**
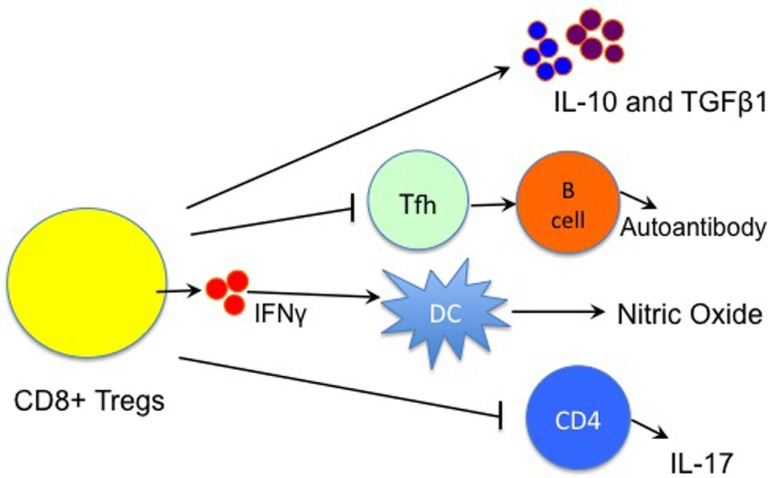
**Immune mechanisms underlying CD8^+^CD122^+^ Treg suppression**. CD8^+^CD122^+^ Treg suppression of immune responses is largely mediated by their production of IL-10 and TGFβ1, whereas IFNγ also plays a role in the suppression by inducing the local production of nitric oxide (NO). They also inhibit the function of T follicular helper (Tfh) cells, resulting in the suppression of autoantibody production by B cells. Moreover, CD8^+^CD122^+^ Tregs suppress autoimmune responses via regulating IL-17 production by CD4^+^ T cells. (Lines with an arrow indicate “producing” or “promoting,” whereas lines with a vertical bar imply “suppressing”).

Although CD8^+^CD122^+^ Tregs have been shown to suppress both alloimmune and autoimmune responses in animal models, their mechanisms of action are not fully understood. Endharti et al. have shown that CD8^+^CD122^+^ Tregs suppress proliferation of conventional CD8^+^ T-cells by producing IL-10 *in vitro* (15). Later on, both IL-10 and TGFβ1 produced by memory CD8^+^CD122^+^ Tregs were reportedly responsible for their suppressive activities (15, 23, 24, 41), as shown in Figure 1. CD8^+^CD122^+^ Tregs recognized conventional T cells via the interaction of MHC class I–αβ TCR and suppressed T-cell function by producing IL-10 (41). We demonstrated that prolongation of allograft survival by IL-10-deficient CD8^+^CD122^+^ Tregs was largely diminished (24). Interestingly, both PD-1 and CD28 signaling pathways on CD8^+^CD122^+^ Tregs were required for their optimal production of IL-10 (24). Moreover, CD8^+^CD122^+^ Tregs released both IFNγ and TGFβ1 that suppressed CD4^+^ T cell activation (22). Krupnick et al. revealed that CCR7 expression on central memory CD8^+^ T-cells was critical for the formation of stable synapses with antigen-presenting cells, which resulted in the production of IFN-γ. The latter in turn induced nitric oxide, which downregulated alloimmune responses and suppressed lung allograft rejection (25). Importantly, CD8^+^CD122^+^ Tregs ameliorated EAE via inhibiting IL-17 production by CD4^+^ effector T-cells, and their suppressive function was dependent on IL-15 (38). On the other hand, disruption of the inhibitory interaction between CD8^+^CD122^+^ T-cells and their target Qa-1^+^ follicular T-helper (Tfh) cells resulted in the development of a lethal systemic-lupus-erythematosus-like autoimmune disease that was dependent on autoantibodies (42). Despite those findings, more studies are required to fully elucidate mechanisms underlying CD8^+^CD122^+^ Treg suppression. Fully understanding their mechanisms of action will help design effective approaches to treating allograft rejection and autoimmune diseases.

## Concluding Remarks

Memory CD8^+^CD122^+^ T-cells have previously been considered to be antigen-specific memory T-cells (8–10). Especially, they exhibit central memory T-cell phenotypes. Mounting evidence has shown that they are also Tregs. Therefore, it is important to define how and when they act as memory versus Tregs. It has been known that abundant PD-1 expression on CD8^+^ T-cells represents their status of exhaustion during chronic viral infection, while PD-1 blockade restores their immune responses (43–46). Recent studies have also shown that T cells undergoing homeostatic proliferation consist of both PD-1^+^ and PD-1^−^ components. It was PD-1^+^ subset of the T-cells that were dysfunctional (47). We have determined if CD8^+^CD122^+^ T-cells also contain both PD-1^+^ and PD-1^−^ subsets. We found that CD8^+^CD122^+^ T-cells contained a subset of PD-1^+^ cell population that actually suppressed allograft rejection, while donor-specific PD-1^−^CD8^+^CD122^+^ T-cells were memory T-cells (24). Hence, PD-1 marker can be utilized to identify murine CD8^+^CD122^+^ Tregs. On the other hand, we have found that “bystander” central memory CD8^+^ T-cells, which do not recognize a specific allograft, are true Tregs (23), whereas others have also shown that memory-like CD122^+^CD8^+^CD38^+^ T-cells suppress CD4^+^ T cell activation in an antigen-independent fashion, indicating that CD8^+^CD122^+^ Tregs act in an antigen-non-specific manner.

Murine CD8^+^CD122^+^ Tregs are naturally arising and exhibit a distribution pattern of two age-related phases. It is unclear if they can be induced from naïve CD8^+^ T-cells. Naturally occurring CD8^+^CD122^+^ Tregs can suppress both autoimmunity and alloimmunity in many animal models of diseases. Mechanisms underlying CD8^+^CD122^+^ Treg suppression mainly include their production of IL-10, TGFβ1, IFNγ, etc. Although IL-15 drives the homeostatic expansion of murine CD8^+^CD122^+^ Tregs, it remains unknown what drives homeostatic proliferation of human CD8^+^CXCR3^+^ Tregs that do not express CD122, although both Tregs exhibit the phenotypes of central memory T-cells. Further studies are warranted to determine if human CD8^+^CXCR3^+^ Tregs can exert suppressive function *in vivo*.

## Author Contributions

JL collected literature and wrote a part of manuscript; DC and GDN edited the manuscript; and ZD wrote the review manuscript.

## Conflict of Interest Statement

The authors declare that the research was conducted in the absence of any commercial or financial relationships that could be construed as a potential conflict of interest.

## Abbreviations

EAE, experimental autoimmune encephalomyelitis; NO, nitric oxide; PD-1, programed death-1; Treg, regulatory T cell.
